# Supplementary material for: Discovery of Novel 3,4-Dihydro-2(1H)-Quinolinone Sulfonamide Derivatives as New Tubulin Polymerization Inhibitors with Anti-Cancer Activity
Source: Molecules. 2022 Feb 24;27(5):1537. doi: 10.3390/molecules27051537 (PMC8911884; doi:10.3390/molecules27051537)
Supplement: Supplementary file 1 [file molecules-27-01537-s001.zip › molecules-1446327-supplementary.pdf]

Synthesis, Evaluation, and Mechanism Study of Quinoline-sulfonamide Derivatives Exerting Effffective Antitumor Activity Through Microtubule Destabilization

Juan Ma[1], Guo-Hua Gong[2,3]*

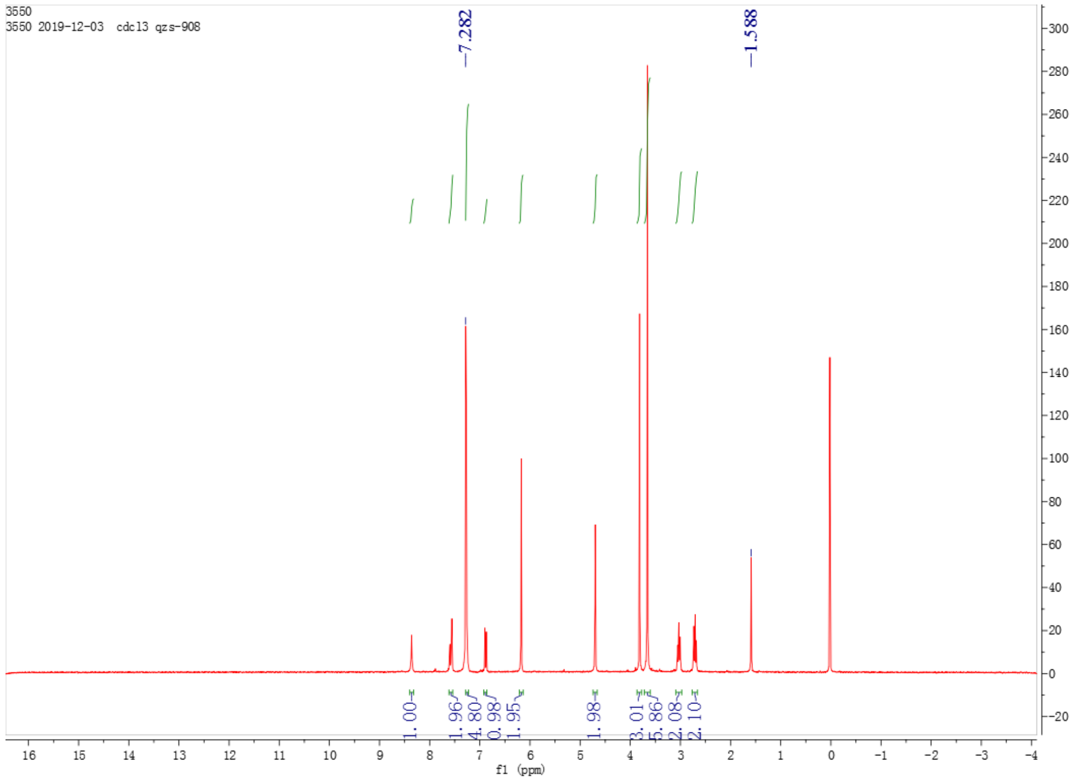

Figure S1. The ^{1}H NMR compound D1

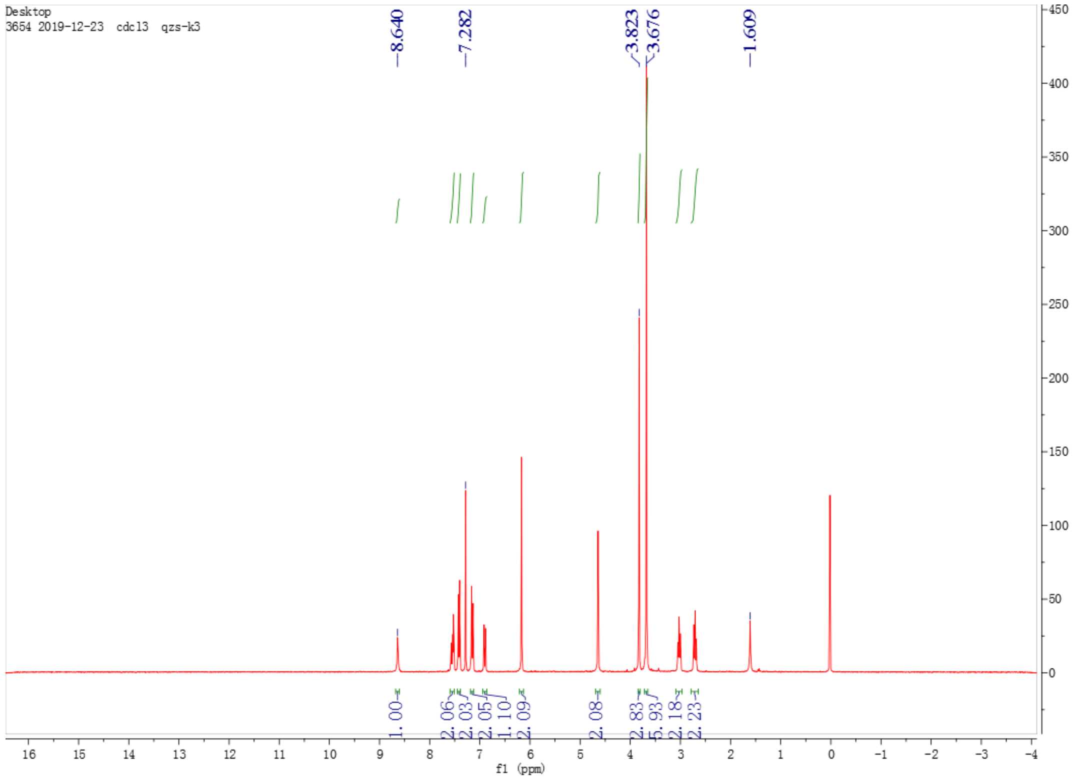

Figure S2. The ^{1}H NMR compound D2

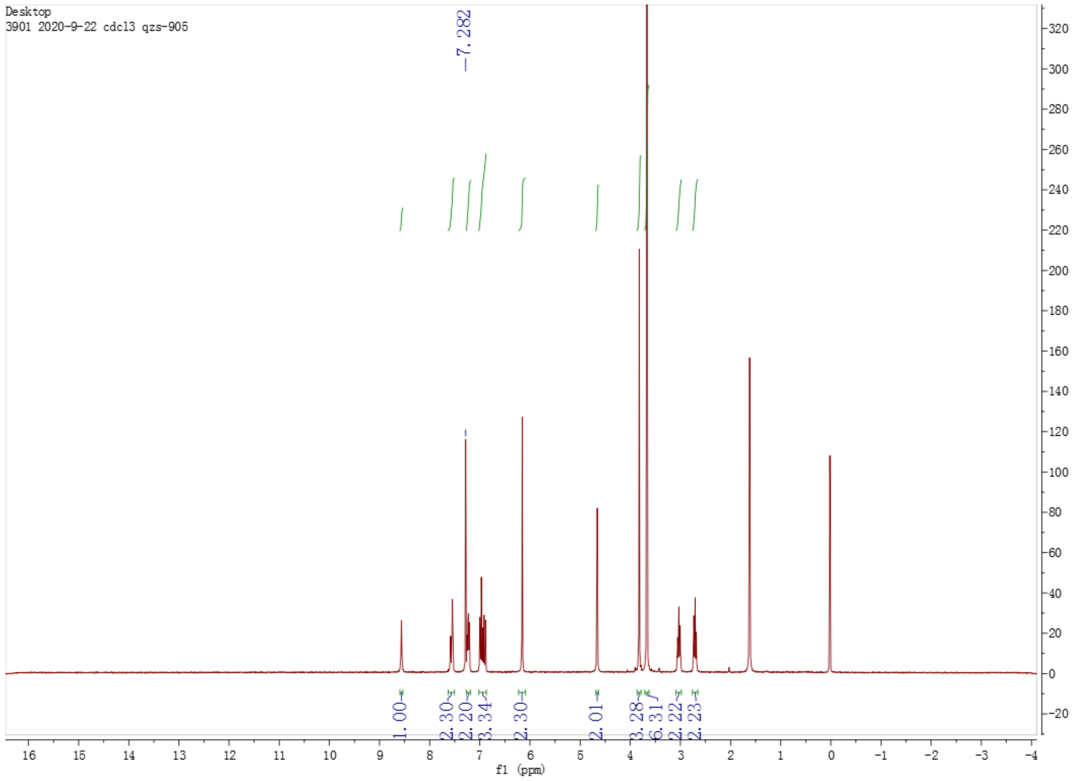

Figure S3. The ^{1}H NMR compound D3

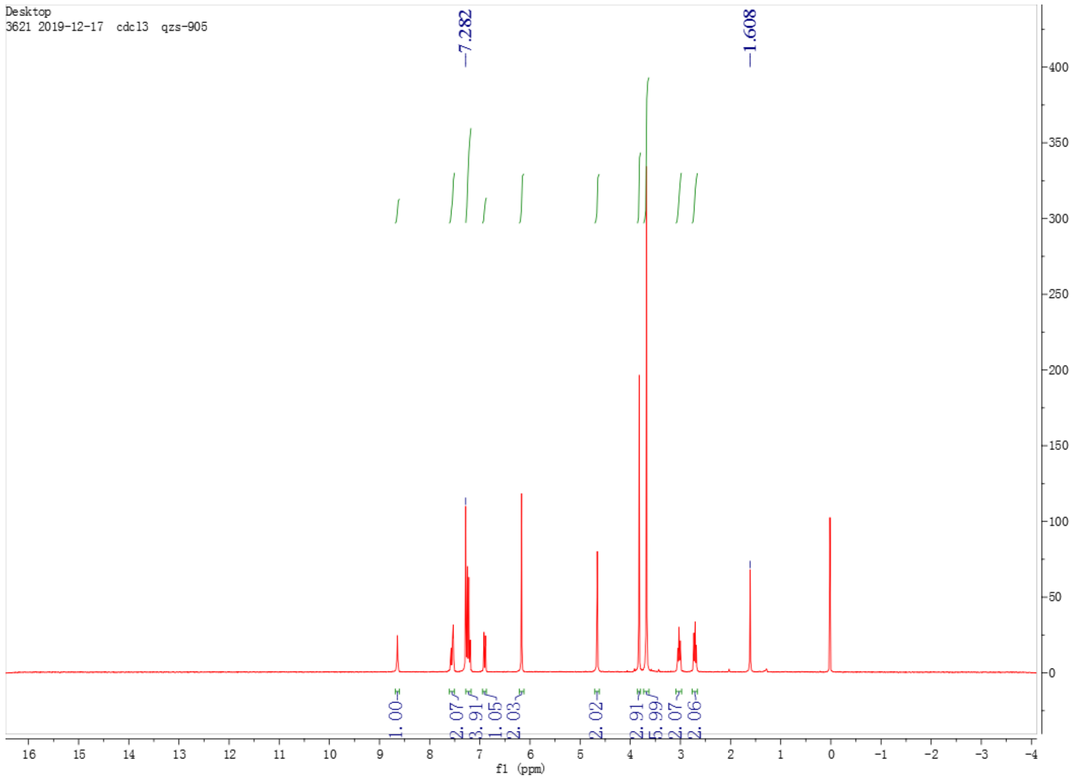

Figure S4. The ¹H NMR compound D4

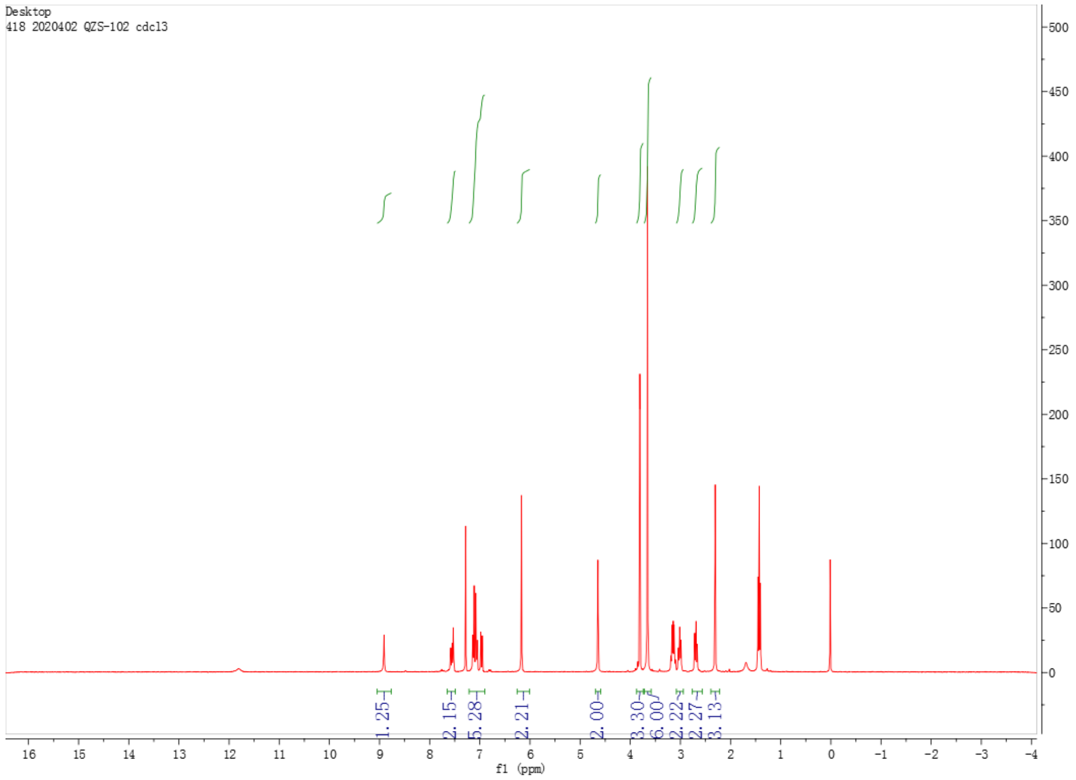

Figure S5. The ¹H NMR compound D5

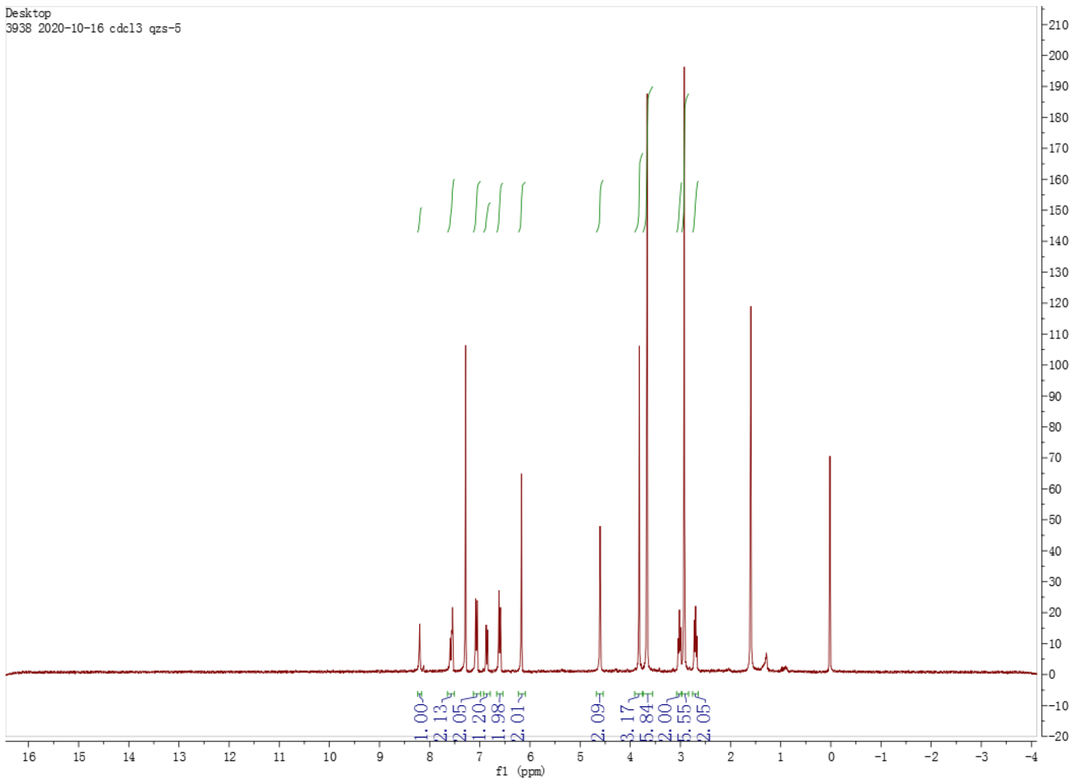

Figure S6. The ^{1}H NMR compound D6

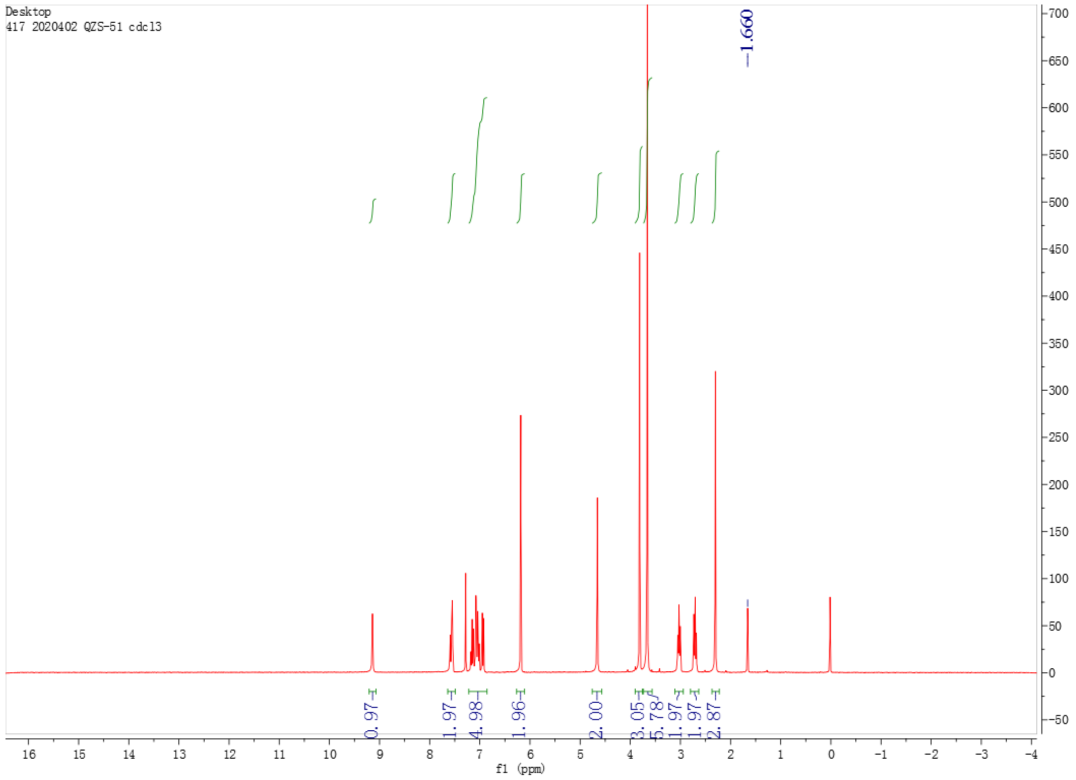

Figure S7. The ^{1}H NMR compound D7

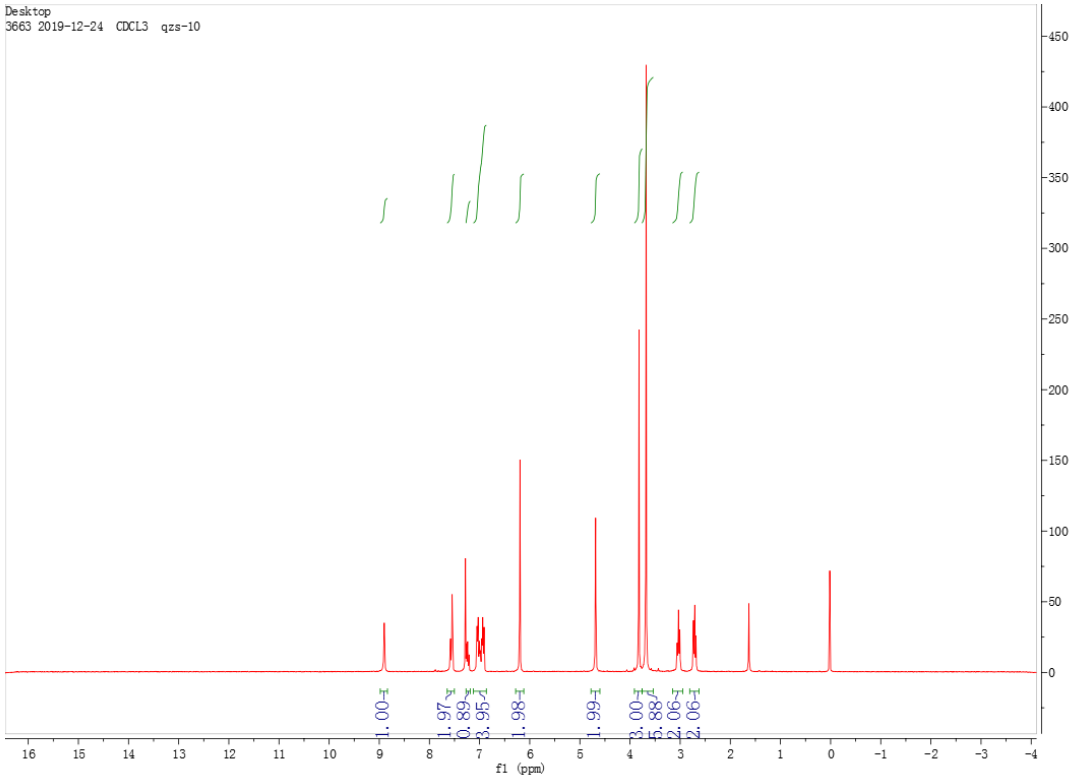

Figure S8. The ^{1}H NMR compound D8

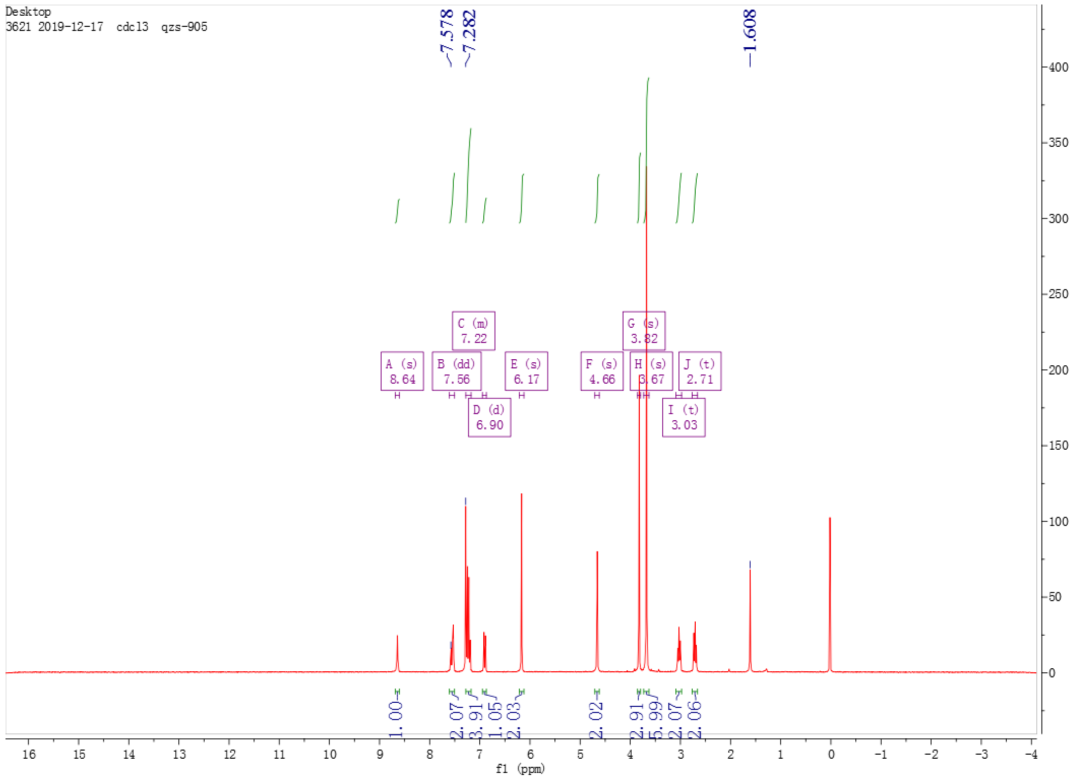

Figure S9. The ^{1}H NMR compound D9

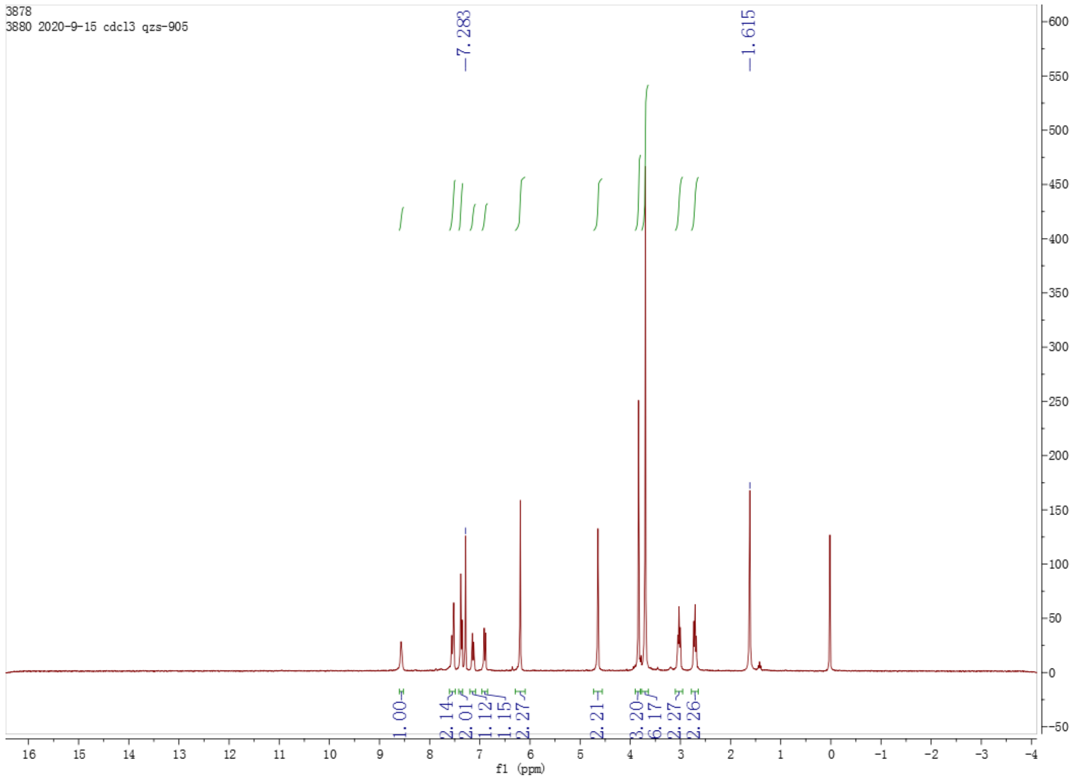

Figure S10. The ^{1}H NMR compound D10

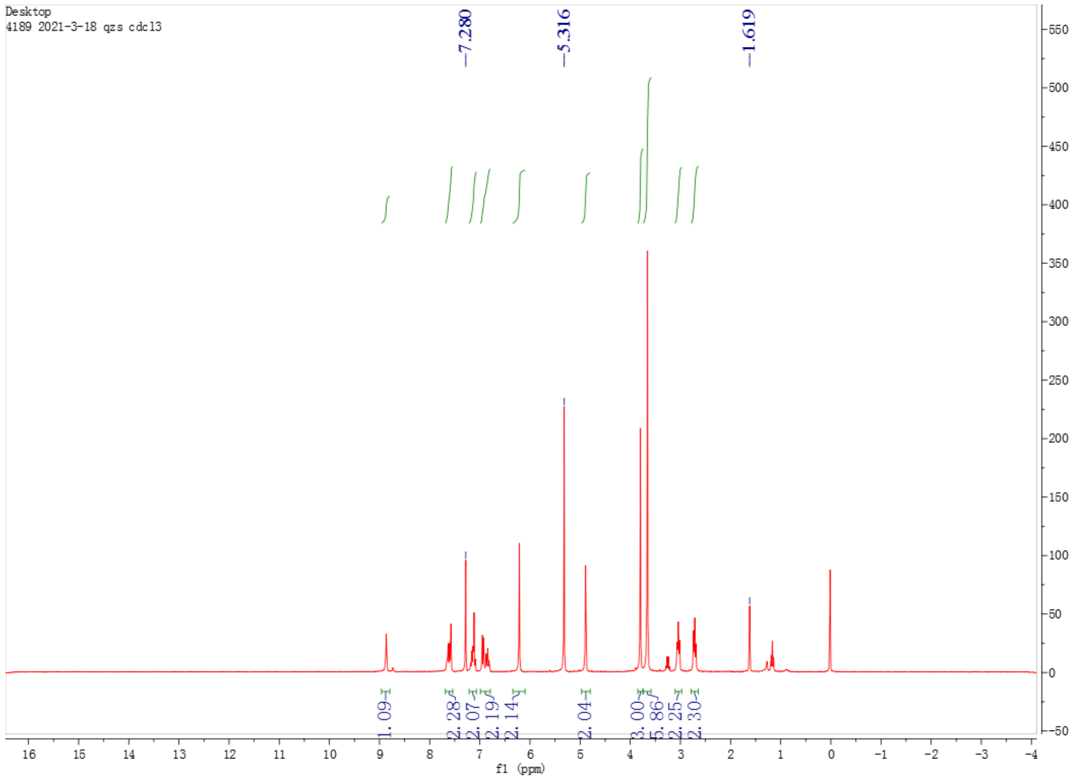

Figure S11. The ^{1}H NMR compound D11

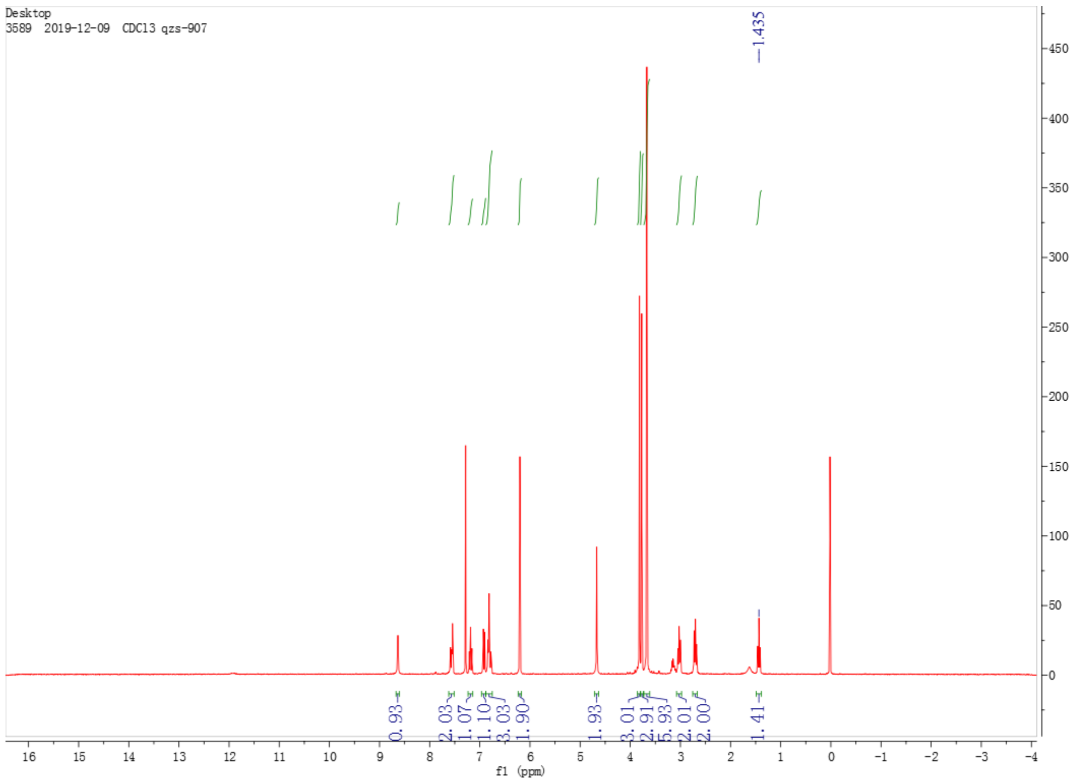

Figure S12. The ^{1}H NMR compound D12

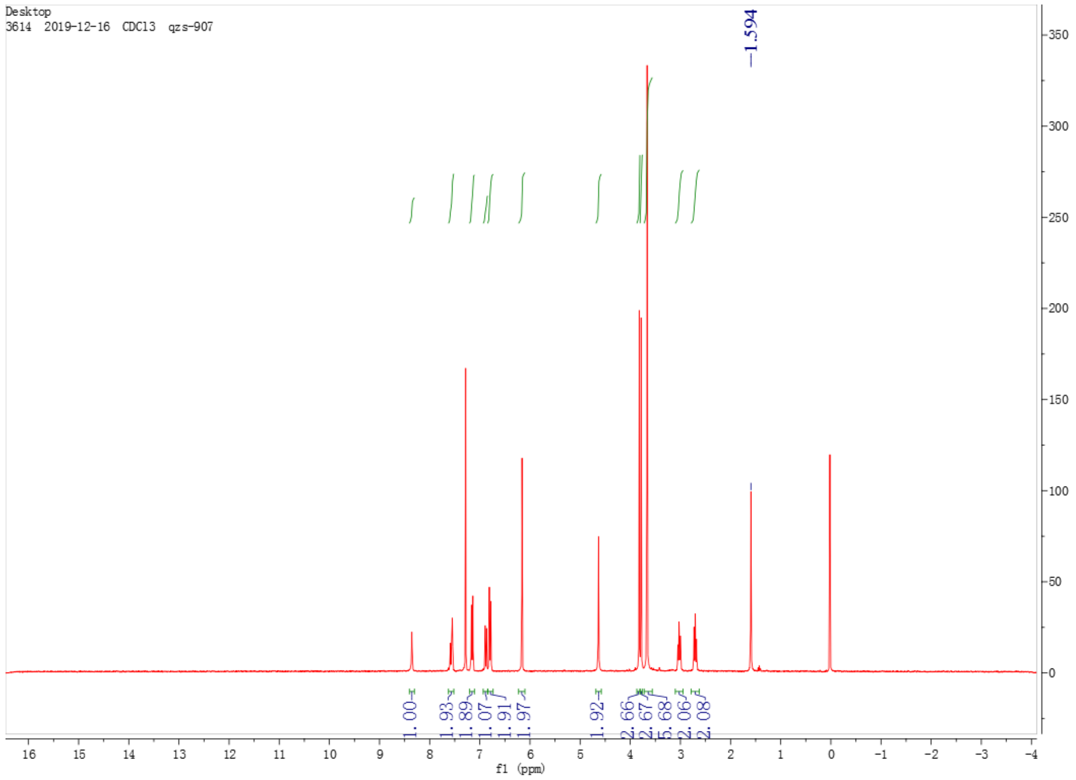

Figure S13. The ^{1}H NMR compound D13

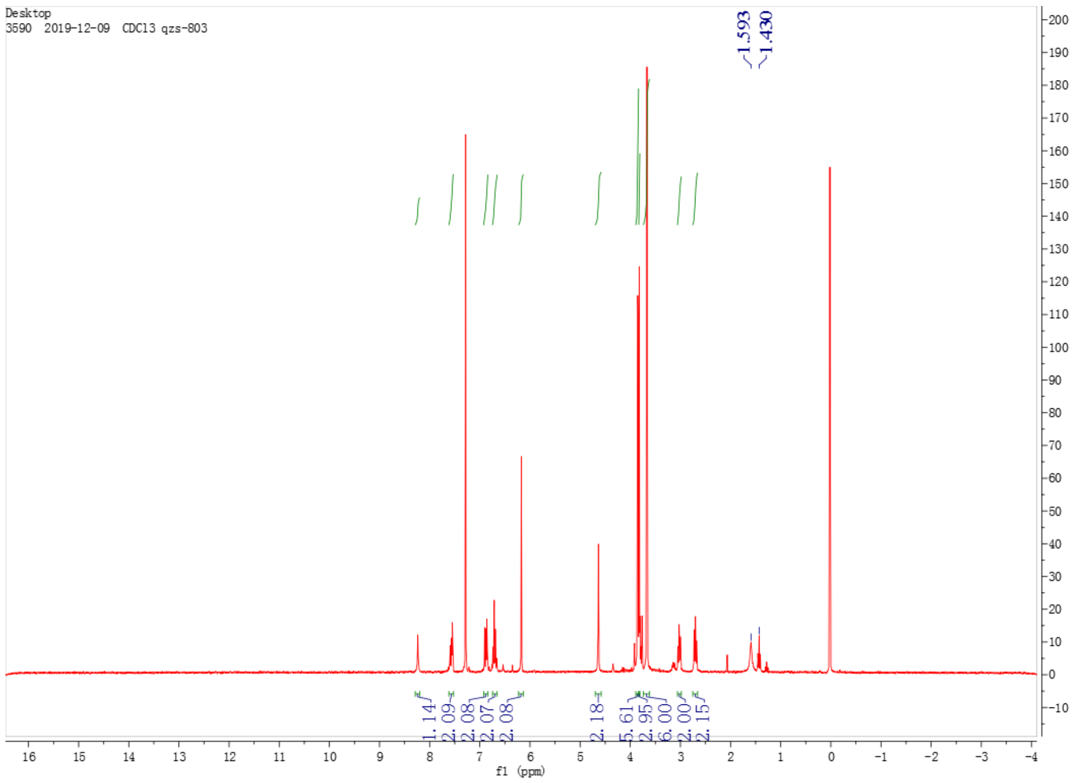

Figure S14. The ^{1}H NMR compound D14

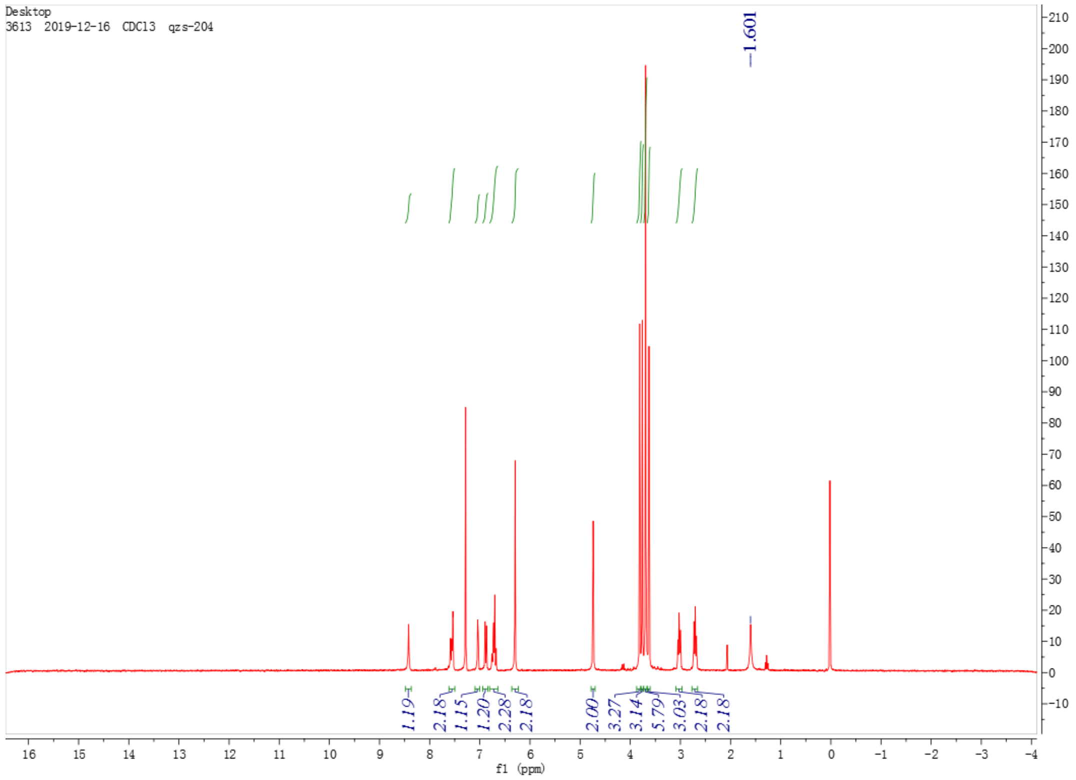

Figure S15. The ^{1}H NMR compound D15

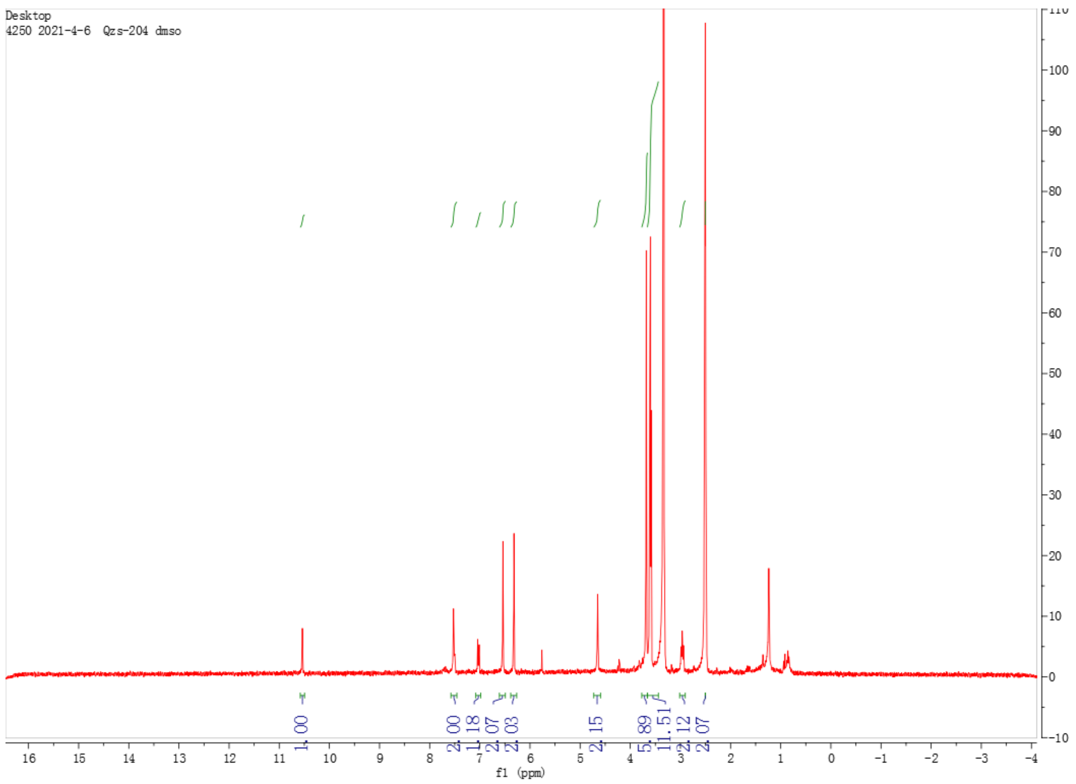

Figure S16. The ^{1}H NMR compound D16
